# Supplementary material for: Molecular mechanism of light-driven sodium pumping
Source: Nat Commun. 2020 May 1;11:2137. doi: 10.1038/s41467-020-16032-y (PMC7195465; doi:10.1038/s41467-020-16032-y)
Supplement: Supplementary file 1 — Supplementary Information [file 41467_2020_16032_MOESM1_ESM.pdf]

Supplementary Information

Molecular mechanism of light-driven sodium pumping

Kovalev K. et al.

Supplementary Discussion

Key role of S-N-D triad in ion translocation.

The S70-N112-D116 (S-N-D) triad, which is completely conserved within NaRs and comprises the core of the Na^+ binding site in the middle part of the protein, is very similar in respect to the residues composition and their relative location to the central gates (CGs) of channelrhodopsins (Supplementary Fig. 15). Indeed, the CGs of the native cation channelrhodopsin-2 (*Cr*ChR2)[1], chimeric cation channelrhodopsin (C1C2)[2] and also natural anion channelrhodopsin (*Gt*ACR1)[3,4] are composed of the S63-N258-E90, S102-N297-E129 and S43-N239-E68 triads (Supplementary Fig. 15). Although the residues are located in the different helices in comparison to the KR2, they form similar overall conformation. CGs serve as constriction sites in the central part of the channels, and are important for the ion selectivity[5]. This allows us to suggest that the transient Na^+ binding site is important for KR2 selectivity. It cannot be also excluded that CGs may act as transient binding sites in channelrhodopsins during ion translocation.

Moreover, in 2015 the evolutional relationship of microbial light-driven Na^+ pumps and class A G protein-coupled receptors (GPCRs) was studied, based on the existing structures of the ground state of KR2[6]. Presented here structure of the O-state with Na^+ bound inside the protein supports the high similarity of the Na^+ binding sites in KR2 and GPCRs. In particular, in both cases the sites are located near helices B and C (TM2 and TM3 in GPCRs), and formed by the similar to the S-N-D triad of KR2 set of residues (D95, N131 and S135 in case of human δ-opioid receptor) (Supplementary Fig. 16). This opens the way for more accurate analysis of the interconnection between two highly important families.

Last but not least, the Na^+ binding site inside KR2 is also very similar to that of another type of Na^+-transporting proteins - Na^+ ATP synthases. For instance, it is almost identical to that of the c11-ring of the Na^+ ATP synthase from *Ilyobacter tartaricus* (Supplementary Fig. 16).

The second Na^+ identified at the KR2 surface in the ground state

Recently we solved crystal structure of KR2 pentameric Na^+-pumping state under physiological conditions at 2.2 Å resolution[7]. In the frame of present work, we improved the resolution of the

model to 2 Å. Therefore, we present here more complete structure of the KR2 resting state at 2 Å (Supplementary Table 1).

While the inner region of the protein protomers is the same as previously reported, we identified numerous of water molecules at the KR2 surface. Moreover, we observe a second Na^+ binding site at the pentamer surface (Supplementary Fig. 17). The additional Na^+ is coordinated by the main chain oxygen of S100 residue and 5 water molecules with the mean Na-O distance of 2.5 Å (Supplementary Fig. 17). Notably, the Na^+ is located close to the putative ion release cavity 2 (pIRC2). As the Na^+ was not identified at lower resolution, and its B-factor is considerably higher than that of the previously reported Na^+ (52 and 21 Å, respectively), we suggest that this ion may play a key role in the relay mechanism of Na^+ release from the pIRC2 to the extracellular space (Supplementary Fig. 17). Indeed, the loosely bound Na^+ may be released to the bulk from the surface upon O-to-ground transition and substituted by the ion transported in the current cycle from the pIRC2.

Crystal structure of the ground and the O-states of KR2 at room temperature

In order to check that freezing does not affect the KR2 conformations in the ground and the O-state we collected diffraction data on KR2 at 293 K using single crystals placed in the stream of humid air (relative humidity of 85%) and solved the structures of the dark (ground) and illuminated (49% ground : 51% O-state) at 2.5 and 2.6 Å, respectively. We also used serial millisecond crystallography (SMX) approach to collect the data at 2.5 Å on the steady-state activated KR2 using microcrystals of the protein injected into the X-ray beam of the synchrotron source in the stream of mesophase. The data allowed us to solve the steady-state-SMX activated structure of KR2 at 2.7 Å. To activate the proteins in crystals, we illuminated them continuously by 532 nm laser during X-ray data collection. This approach allows accumulation of the dominant intermediate of protein photocycle, which in the case of KR2 is the O-state. Analysis of the electron density maps and occupancies refinement indicated that such procedure results in the 51 and 50 % occupancy of the O-state in case of single-crystal and serial millisecond crystallography approaches, respectively.

Overall, the structures of the ground and the O-states of KR2 at 100 and 293 K are nearly identical (Supplementary Fig. 18, 19). The RMSD between the structures of the ground state of

KR2 at 100 and 293K is 0.2 Å, and between those of the O-state it is also 0.2 Å. Comparison of the KR2 structures identified slight shifts of the positions of the E-F and F-G loops and also cytoplasmic parts of helices E and F by only 0.4 Å (Supplementary Fig. 18). The orientations of all key residues and the arrangement of water molecules inside the protein are similar in models at 100 and 293K (Supplementary Fig. 19).

Double conformation of the $RSBH^+$ in D116N mutant

The electron densities around retinal and K255 in the D116N mutant suggest the coexistence of two alternative orientations of the $RSBH^+$ (Supplementary Fig. 20). In one of them, similar to the O-state and 'compact' conformation of KR2, $RSBH^+$ is pointed towards N116. However, there is no hydrogen bond between them and the distance between $RSBH^+$ and N116 is 4.3 Å. Likely, there is a hydrogen bond to N112 in this conformation. In the second conformation, $RSBH^+$ is shifted closer to D251 (3.1 Å) and may form a hydrogen bond with the residue (Supplementary Fig. 6D, 21). In both conformations, retinal remains in the all-*trans* configuration (Supplementary Fig. 20). Importantly, such organization of the $RSBH^+$ region is in line with the existing spectroscopic and nuclear magnetic resonance (NMR) data on D116N mutant. Indeed, it was shown, that the hydrogen bond between the $RSBH^+$ and $D116^-$ exists in only a fraction of this KR2 variant. Moreover, as it was demonstrated by the NMR studies, the $RSBH^+$ is in a multiple conformations in D116N[8]. Therefore, we refined our crystallographic data on the mutant with the both alternative $RSBH^+$ orientations in the final model (Supplementary Fig. 20).

The basis of the KR2 pentamer dissociation at acidic pH

We showed previously that the oligomeric state of the KR2 is pH-dependent in the detergent micelles and also in the crystals grown from the lipidic cubic phase[7,9]. KR2 is organized into pentamers at pH values higher than 6-6.5, while the monomers are found at acidic pH. The basis of pentamer disruption at low pH remains unclear. One of the hypotheses is the influence of pH on the rechargeable residues of the oligomerization interface, such as H30. It was demonstrated that pentameric assembly is disturbed in H30K and H30L mutants[7]. However, the H30A mutant remained pentameric[10]. This suggests another mechanism of oligomer dissociation. As it is shown, with the pH decrease not only the pentameric assembly is affected, but also protonation of D116 occurs. For the wild type protein, the shift from the pentameric to monomeric state

appears detergent at pH 5-6, which is also close to the pKa of D116. Consequently, it is natural to suggest that the protonation of D116 may influence the oligomeric state of the protein and lead to the pentamer dissociation. To check the hypothesis, we studied oligomerization of the D116N in detergent micelles using size-exclusion chromatography (SEC). The mutant D116N imitates the protein with protonated D116 at all pH values. We showed that at pH 8.0, where KR2 forms pentamers, D116N is observed in several oligomeric states. Notable portions of both smaller and bigger oligomers are present in the solution. As follows from the analysis of the D116N oligomerization dependence on pH, the smaller oligomers dominate at pH 6, but some intermediate-sized oligomers (smaller than pentamers of the wild type protein) appear as pH is lowered to 4.3 (Supplementary Fig. 21). Altogether, this allows us to suggest that the protonation of D116 and presumably binding of Na^+ near D116 in the O-state affects the oligomeric state of KR2 and is one of the driving forces of the destabilization of the KR2 pentamer. Consequently, this additionally supports the fact that KR2 protomers are distorted in the O-state and pentamerization interface could also be affected. Thus, pentameric assembly not only plays a key role in the organization of the ‘expanded’ conformation, important for the Na^+ release, but also stabilizes the proteins in the O-state with Na^+ bound in close proximity of the $RSBH^+$.

Supplementary References

1. Volkov, O. *et al.* Structural insights into ion conduction by channelrhodopsin 2. *Science (80-.).* **358**, (2017).
2. Kato, H. E. *et al.* Crystal structure of the channelrhodopsin light-gated cation channel. *Nature* (2012). doi:10.1038/nature10870
3. Li, H. *et al.* Crystal structure of a natural light-gated anion channelrhodopsin. *Elife* (2019). doi:10.7554/eLife.41741
4. Kim, Y. S. *et al.* Crystal structure of the natural anion-conducting channelrhodopsin GtACR1. *Nature* (2018). doi:10.1038/s41586-018-0511-6
5. Wietek, J. *et al.* Conversion of channelrhodopsin into a light-gated chloride channel. *Science (80-.).* **344**, 409–412 (2014).
6. Shalaeva, D. N., Galperin, M. Y. & Mulkidjanian, A. Y. Eukaryotic G protein-coupled receptors as descendants of prokaryotic sodium-translocating rhodopsins. *Biol. Direct* (2015). doi:10.1186/s13062-015-0091-4
7. Kovalev, K. *et al.* Structure and mechanisms of sodium-pumping KR2 rhodopsin. *Sci. Adv.* **5**, eaav2671 (2019).
8. Shigeta, A. *et al.* Solid-state nuclear magnetic resonance structural study of the retinal-binding pocket in sodium ion pump rhodopsin. *Biochemistry* (2017). doi:10.1021/acs.biochem.6b00999
9. Gushchin, I. *et al.* Crystal structure of a light-driven sodium pump. *Nat. Struct. Mol. Biol.* **22**, 390–396 (2015).
10. Kaur, J. *et al.* Solid-state NMR analysis of the sodium pump Krokinobacter rhodopsin 2 and its H30A mutant. *J. Struct. Biol.* (2018). doi:10.1016/j.jsb.2018.06.001
11. Liebschner, D. *et al.* Polder maps: Improving OMIT maps by excluding bulk solvent. *Acta Crystallogr. Sect. D Struct. Biol.* (2017). doi:10.1107/S2059798316018210
12. Ho, B. K. & Gruswitz, F. HOLLOW: Generating accurate representations of channel and interior surfaces in molecular structures. *BMC Struct. Biol.* **8**, (2008).
13. Yun, J. H. *et al.* Non-cryogenic structure of a chloride pump provides crucial clues to temperature-dependent channel transport efficiency. *J. Biol. Chem.* (2019). doi:10.1074/jbc.RA118.004038
14. Luecke, H., Schobert, B., Richter, H. T., Cartailler, J. P. & Lanyi, J. K. Structure of bacteriorhodopsin at 1.55 Å resolution. *J. Mol. Biol.* **291**, 899–911 (1999).
15. Volkov, O. *et al.* Structural insights into ion conduction by channelrhodopsin 2. *Science (80-.).* **358**, (2017).

Supplementary Figures

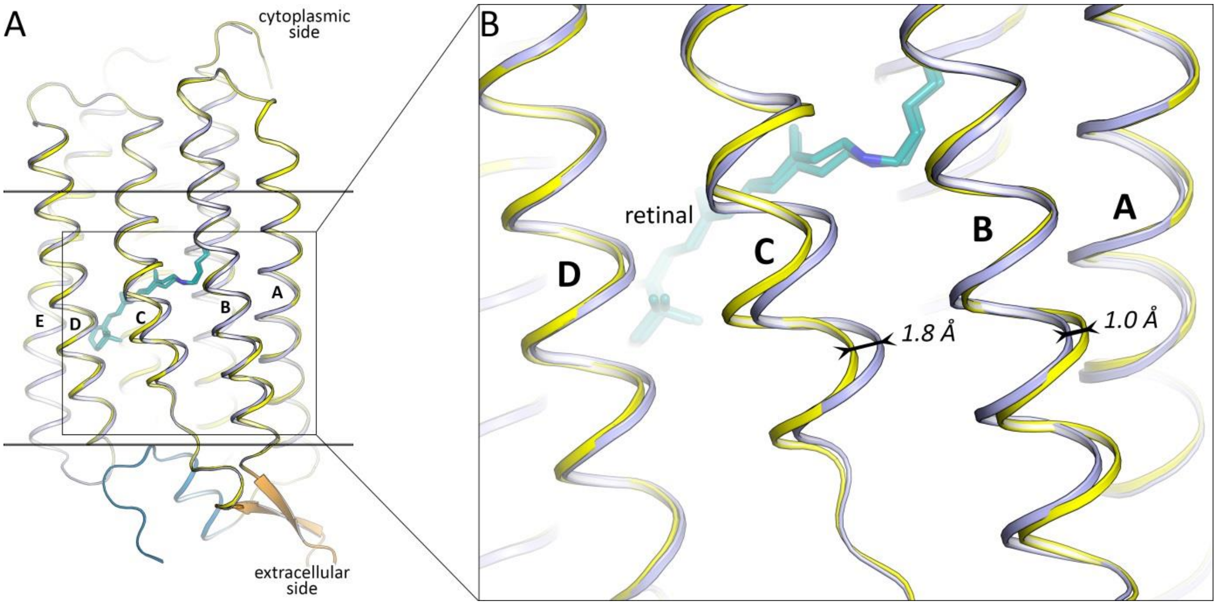

Supplementary Figure 1. Structural alignment of KR2 protomers in the ground (yellow) and the O- (blue) states. A. Overall alignment. N-terminal α-helix and N terminus are colored blue. BC loop, containing the β-sheet, is colored orange. **B.** Enlarged view of the most notable rearrangements in protomer backbone. Retinal cofactor is colored teal. Membrane core boundaries are shown with black lines. Helices are indicated with capital letters. The shifts of extracellular parts of helices B and C are demonstrated with black arrows.

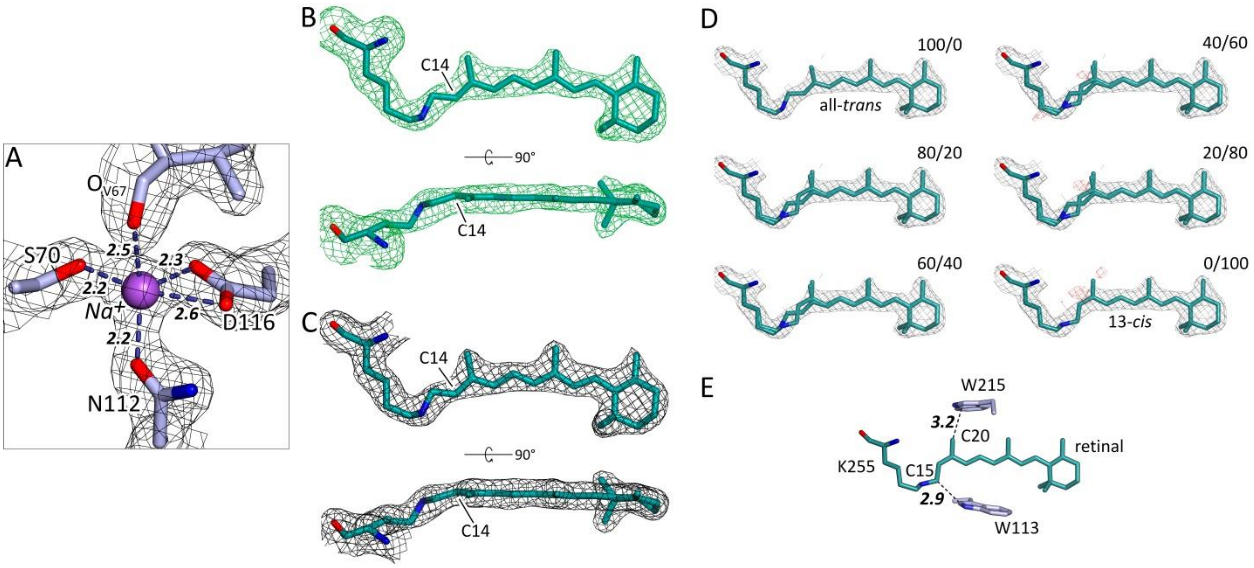

Supplementary Figure 2. Examples of electron densities of the KR2 O-state. A. $2F_o$-F_c electron density maps around sodium ion binding site contoured at the level of 1.5 σ. Sodium ion is shown with violet sphere. Hydrogen bonds, coordinating the sodium ion are shown with blue dashed lines. **B.** Polder[11] maps for K255 and retinal cofactors of all five protomers of KR2 O-state structure. Maps are contoured at the level of 4.0 σ. **C.** $2F_o$-F_c electron density maps around K255 and retinal cofactor contoured at the level of 1.5 σ. **D.** $2F_o$-F_c and F_o-F_c electron density maps built using the data of the O-state of KR2 at 100K at 2.1 Å and 6 models with 100/0, 80/20, 60/40, 40/60, 20/80 and 0/100 proportions of all-*trans*/13-*cis* retinal configurations ratios. $2F_o$-F_c maps are contoured at the level of 1.5 σ and are shown with gray mesh. Difference negative F_o-F_c maps are contoured at the level of 3 σ and are shown with red mesh. **E.** Steric conflict and inadequately short distances between retinal C_{15} and C_{20} atoms to the nearby residues W113 and W215 when fitting the data with 13-*cis* retinal. Retinal and K255 are colored teal. C_{14}, C_{15} and C_{20} atoms of retinal are indicated. The lengths of the hydrogen bonds are shown with bold italic numbers and are in Å.

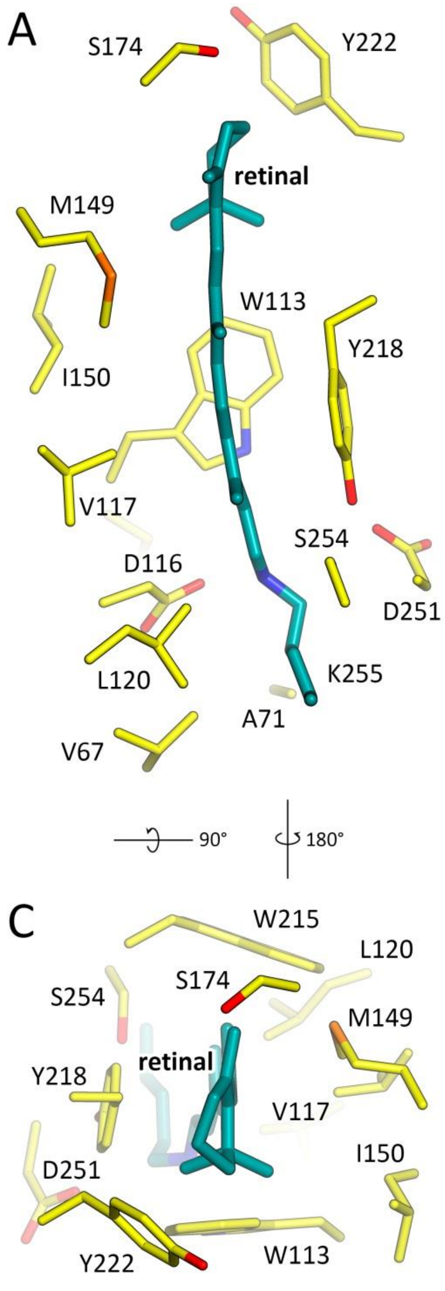

Supplementary Figure 3. Retinal binding pockets of ground (yellow) and O (blue) states. A, B. View from the cytoplasmic side. **C, D.** View from the side of β-ionone ring of the retinal molecule. Retinal cofactor is colored teal.

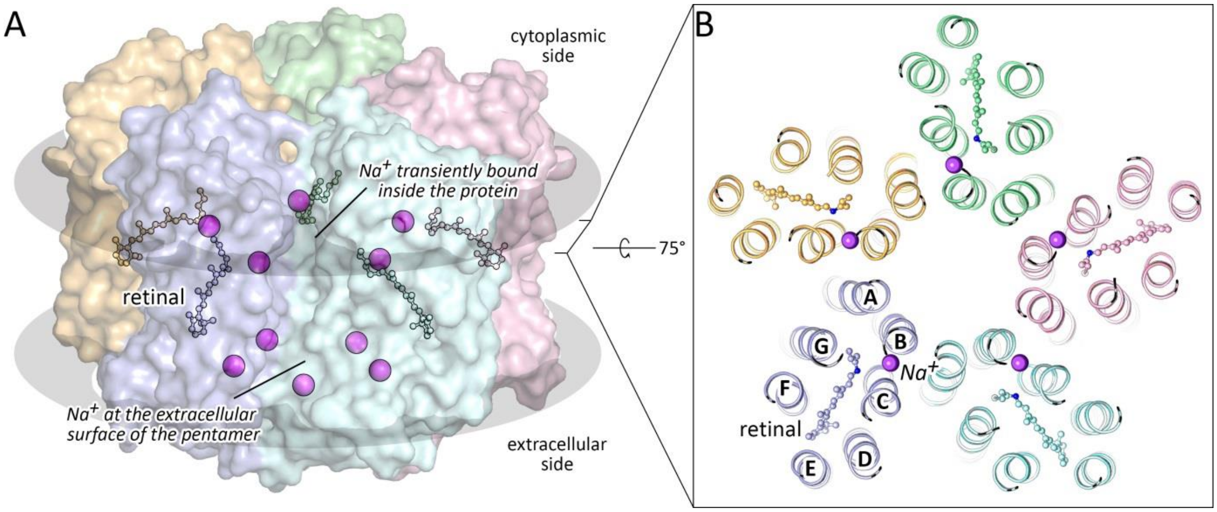

Supplementary Figure 4. KR2 pentamer in the O-state and binding site of Na^+. A. Side view of the KR2 pentamer, shown in surface representation. Membrane core boundaries are shown with black ellipses. **B.** Section view from the cytoplasmic side on the central part of the KR2 pentamer. Sodium ion is placed between helices B and C. Helices are indicated with capital letters. Sodium ions are colored violet. Retinal molecules and covalently bound Lys255 side chains are shown in balls and sticks representation.

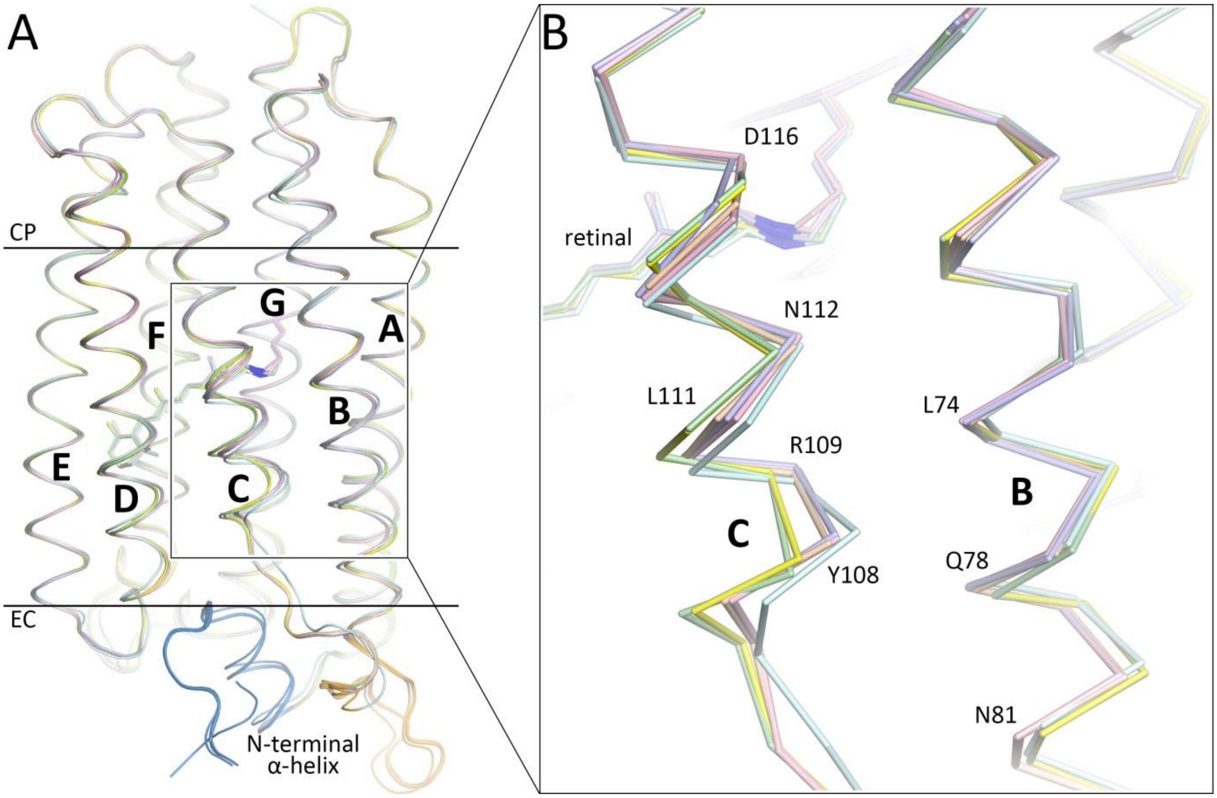

Supplementary Figure 5. Structural alignment of KR2 protomers in different states. A. Overall alignment. N-terminal α-helix and N terminus are colored blue. BC loop, containing the β-sheet, is colored orange. Membrane core boundaries are shown with black lines. **B.** Enlarged view of the most notable rearrangements in protomer backbone. Helices are indicated with capital bold letters. Ground state of KR2 (the 'expanded' conformation, PDB ID: 6REW) is colored yellow. O-state of KR2 (present work) is colored light-blue. KR2-D116N (present work) is colored pink. KR2-H30A (present work) is colored green. Ground state of KR2 (the 'compact' conformation, PDB ID: 4XTN, chain 'I') is colored orange. Ground state of monomeric KR2 (PDB ID: 4XTL) is colored cyan.

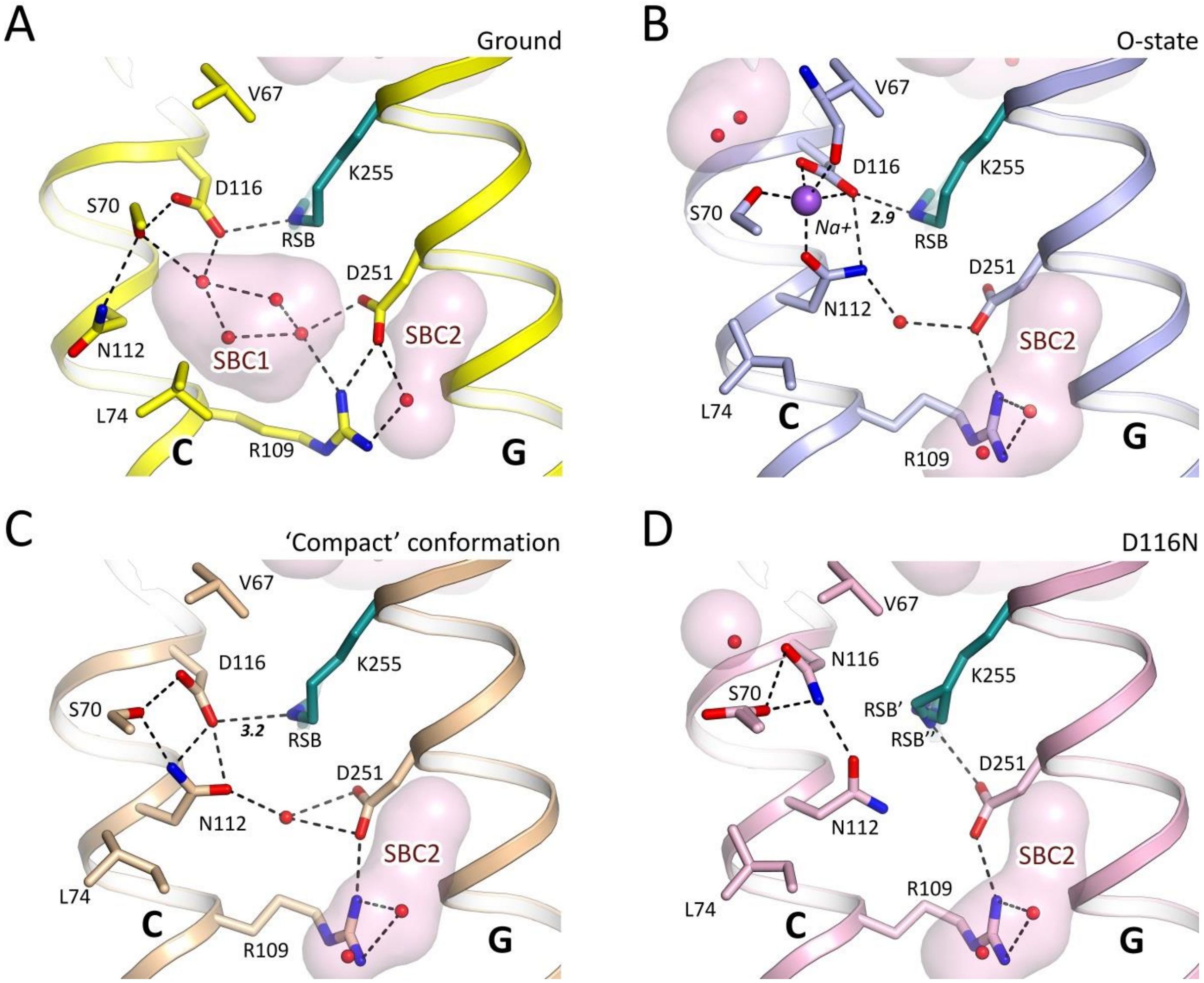

Supplementary Figure 6. RSB region of the KR2 in different states. **A.** Ground state of KR2 (PDB ID: 6REW) **B.** O-state of KR2 (present work). **C.** The 'compact' conformation of KR2 (PDB ID: 4XTN, chain 'I'). **D.** D116N mutant of KR2 (present work). Cavities are calculated using HOLLOW[12] and shown with pink surfaces. Retinal cofactor is colored teal. Water molecules are shown with red spheres. Na^+ is shown with a purple sphere. Hydrogen bonds are shown with black dashed lines. The lengths of the D116-RSB hydrogen bond are shown with bold italic numbers and are in Å. Helices C and G are indicated with capital bold letters. Helices A and B are hidden for clarity.

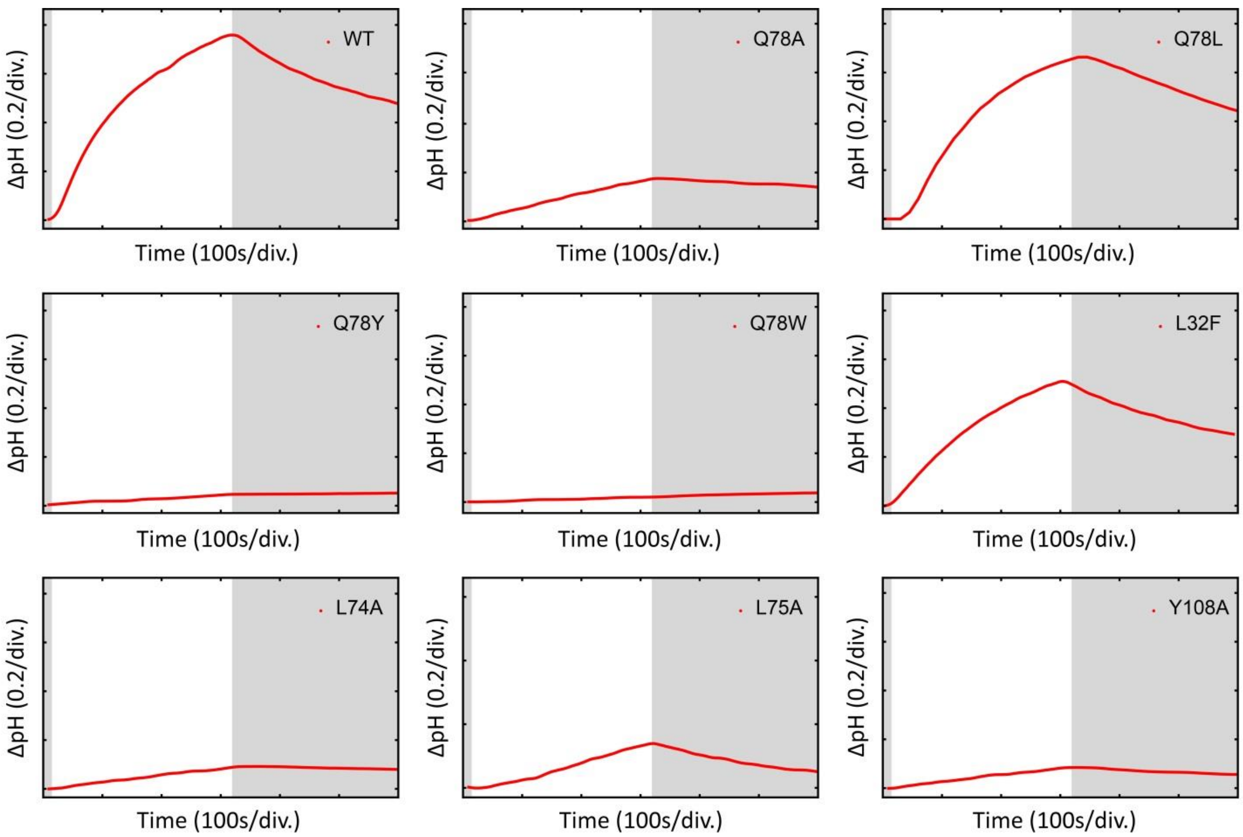

Supplementary Figure 7. *E. coli* activity tests of KR2 and its mutants. pH changes upon illumination in the media containing KR2-expressing *E. coli* cells. The solutions contain 100 mM NaCl and 30 µM CCCP (magenta). The cells were illuminated for 300 s (light area on the plots).

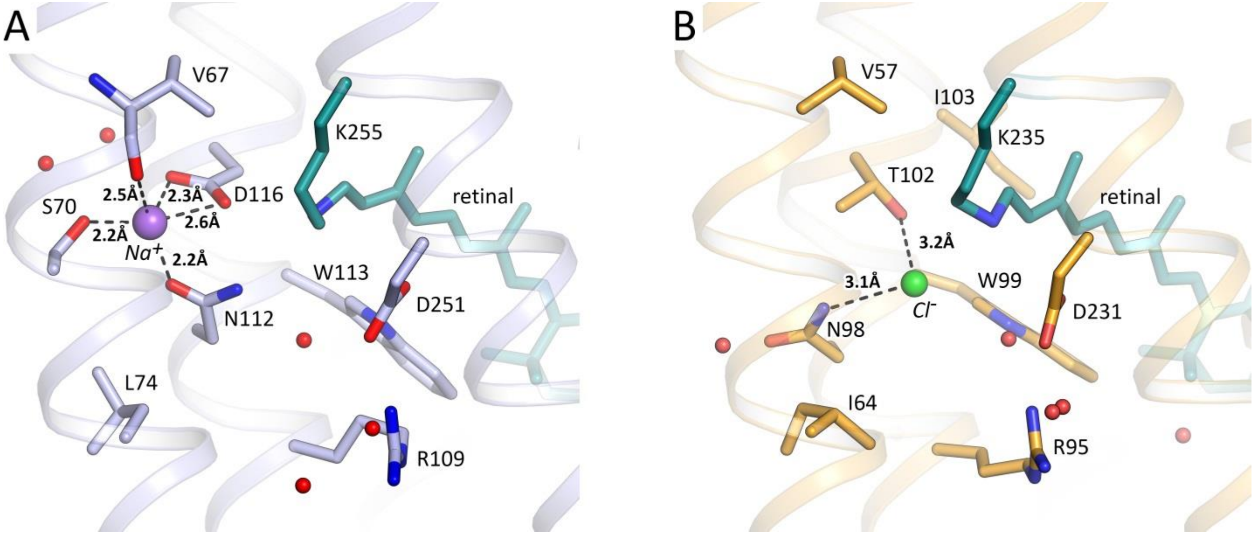

Supplementary Figure 8. Na^+ and Cl^- binding sites of the ion-pumping bacterial rhodopsins. **A.** O-state of KR2 (present work). **B.** Ground state of the chloride-pumping *Nonlabens marinus* S1-08 rhodopsin (PDB ID: 5ZTK[13]). Retinal cofactor is colored teal. Water molecules are shown with red spheres. Na^+ is shown with a purple sphere. Cl^- is shown with a green sphere. Distances are shown with black dashed lines. The length of the D116-RSB hydrogen bond are shown with bold italic numbers and are in Å. Helix A is hidden for clarity.

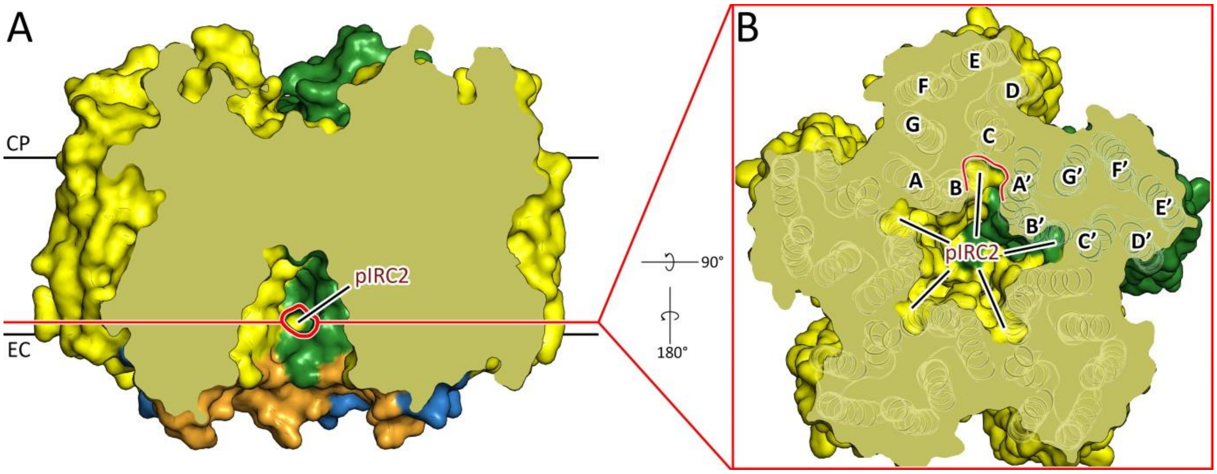

Supplementary Figure 9. Putative ion-release cavity 2 (pIRC2) of the ground state of KR2. A. Side section view of KR2 pentamer. Red ellipse-like shape contours the pore in the protein surface leading to the pIRC2 from the concave aqueous basin formed in the central pore of KR2 pentamer. Membrane core boundaries are shown with black lines. **B.** Section view from the extracellular side at the level of the pIRC2. pIRC2 of each protomer is formed by helices B, C and BC loop and A' from adjacent protomer. The pIRC2 from the section A is also contoured with red line. Helices are signed with capital bold letters.

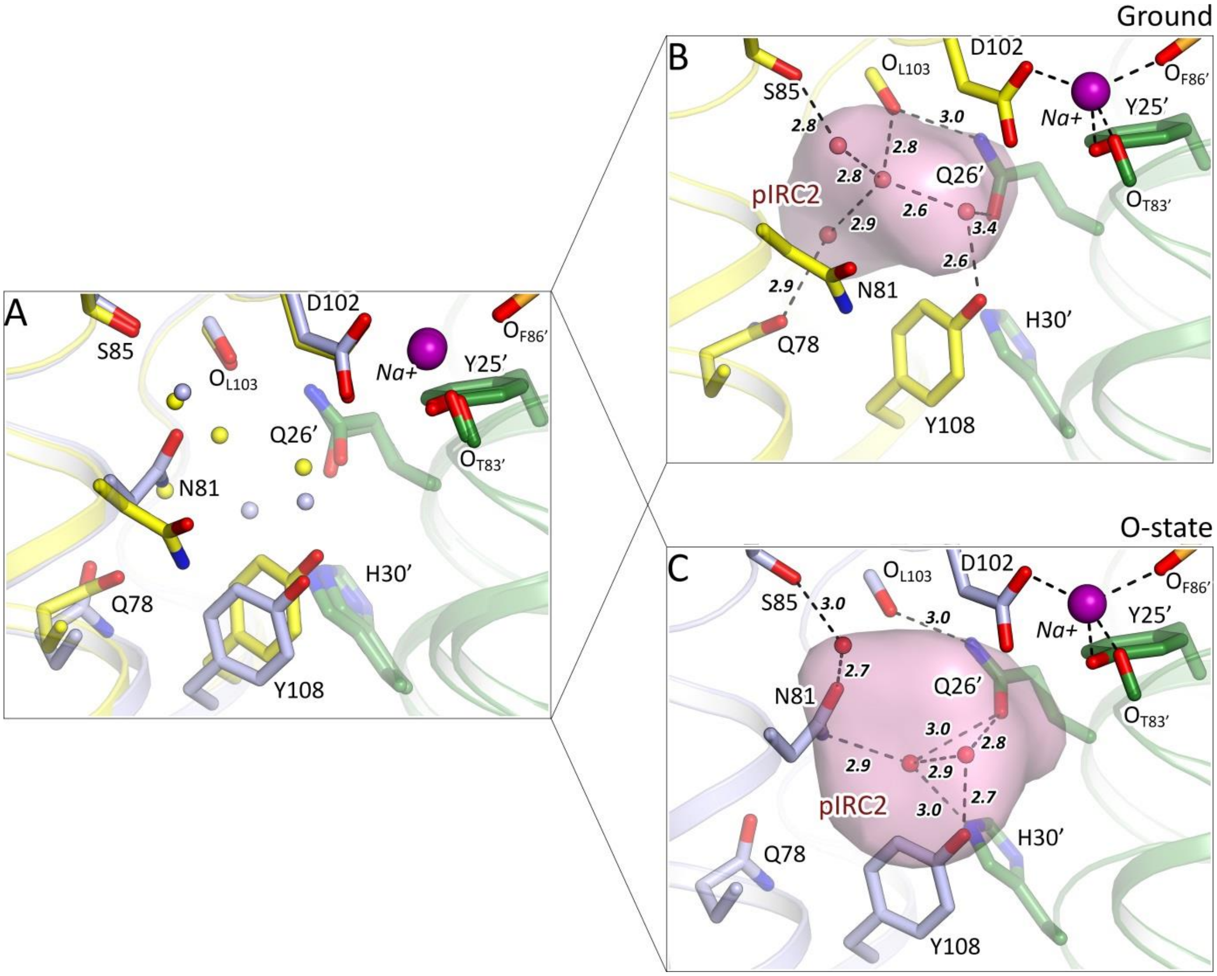

Supplementary Figure 10. Putative ion-release cavity 2 (pIRC2) of the ground and O-states of KR2. A. Structural alignment of the ground (yellow, PDB ID: 6REW) and the O- (blue, present work) states. **B.** Detail view of the pIRC2 in the ground state. **C.** Detail view of the pIRC2 in the O-state. Na^+ at the extracellular surface of KR2 pentamer are shown with purple spheres. Adjacent protomer is colored green. Hydrogen bonds are shown with black dashed lines. The length of the hydrogen bonds are shown with bold italic numbers and are in Å. Water molecules are shown with small spheres and colored yellow and blue in section A and red in sections B and C.

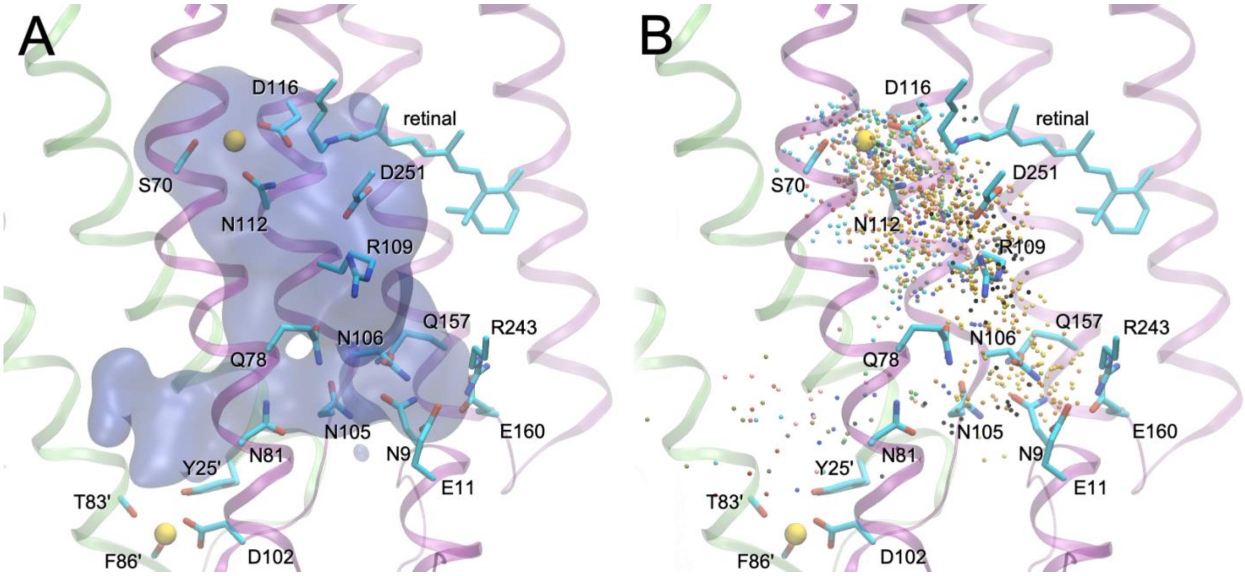

Supplementary Figure 11. Simulated trajectories of Na^+ release identified by molecular dynamics simulations. **A.** Density surface corresponding to the volume accessible to Na^+. **B.** Positions of sodium taken every 100 ps. Each trajectory is shown in a different color; trajectories where sodium exits via pIRC1 are shown in yellow and orange. Two protomers of KR2 pentamer are rendered as purple and green ribbons. X-ray orientations of key amino acid sidechains are shown.

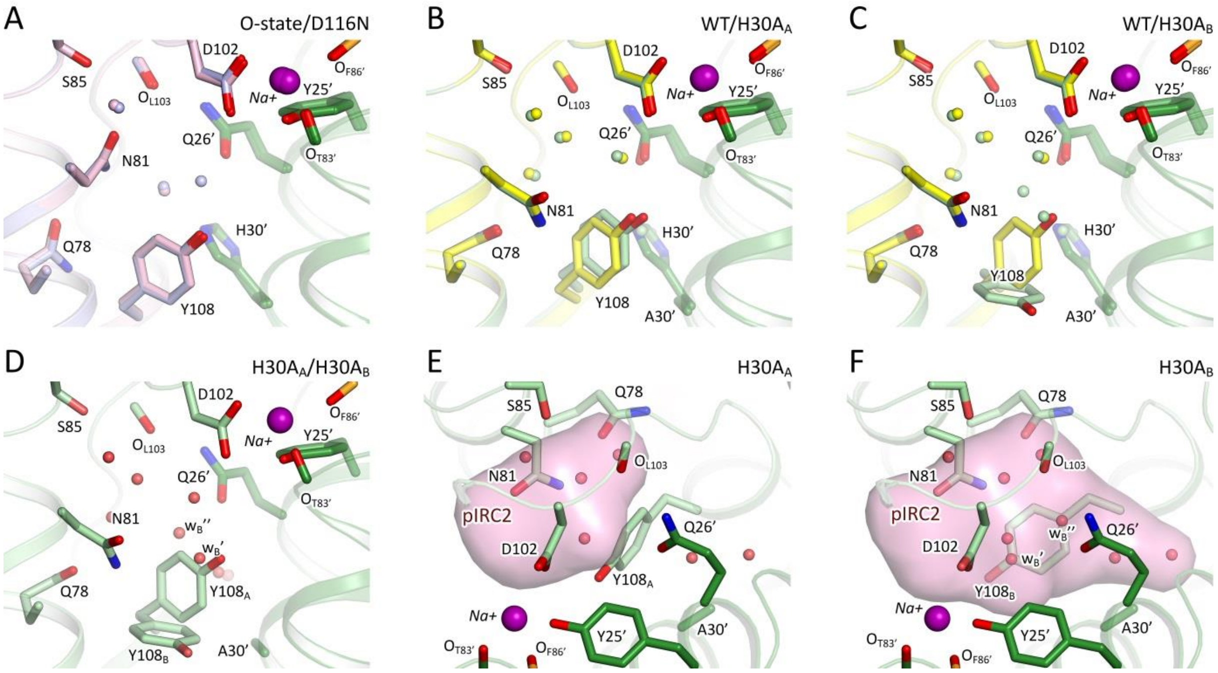

Supplementary Figure 12. Putative ion-release cavity 2 (pIRC2) of the D116N and H30A mutants of KR2. A. Structural alignment of the O-state of the WT KR2 (blue, present work) and the D116N ground state (pink, present work). The conformations are nearly identical. **B.** Structural alignment of the ground states of the WT KR2 (yellow, PDB ID: 6REW) and conformation A of H30A mutant ($H30A_A$, light green, present work). **C.** Structural alignment of the ground states of the WT KR2 (yellow, PDB ID: 6REW) and conformation B of H30A mutant ($H30A_B$, light green, present work). **D.** Structural alignment of the ground states of the conformations A and B of H30A mutant. Additional water molecules, appearing in the structure of the $H30A_B$ are identified as w_B' and w_B''. **E.** Detail view of the pIRC2 of the $H30A_A$. **F.** Detail view of the pIRC2 of the $H30A_B$. pIRC2 is notably enlarged in the $H30A_B$. Na^+ at the extracellular surface of KR2 pentamer are shown with purple spheres. Adjacent protomer is colored dark green. Water molecules are shown with small spheres and colored blue and pink in section A, yellow and light green in sections B and C and red in sections D-F.

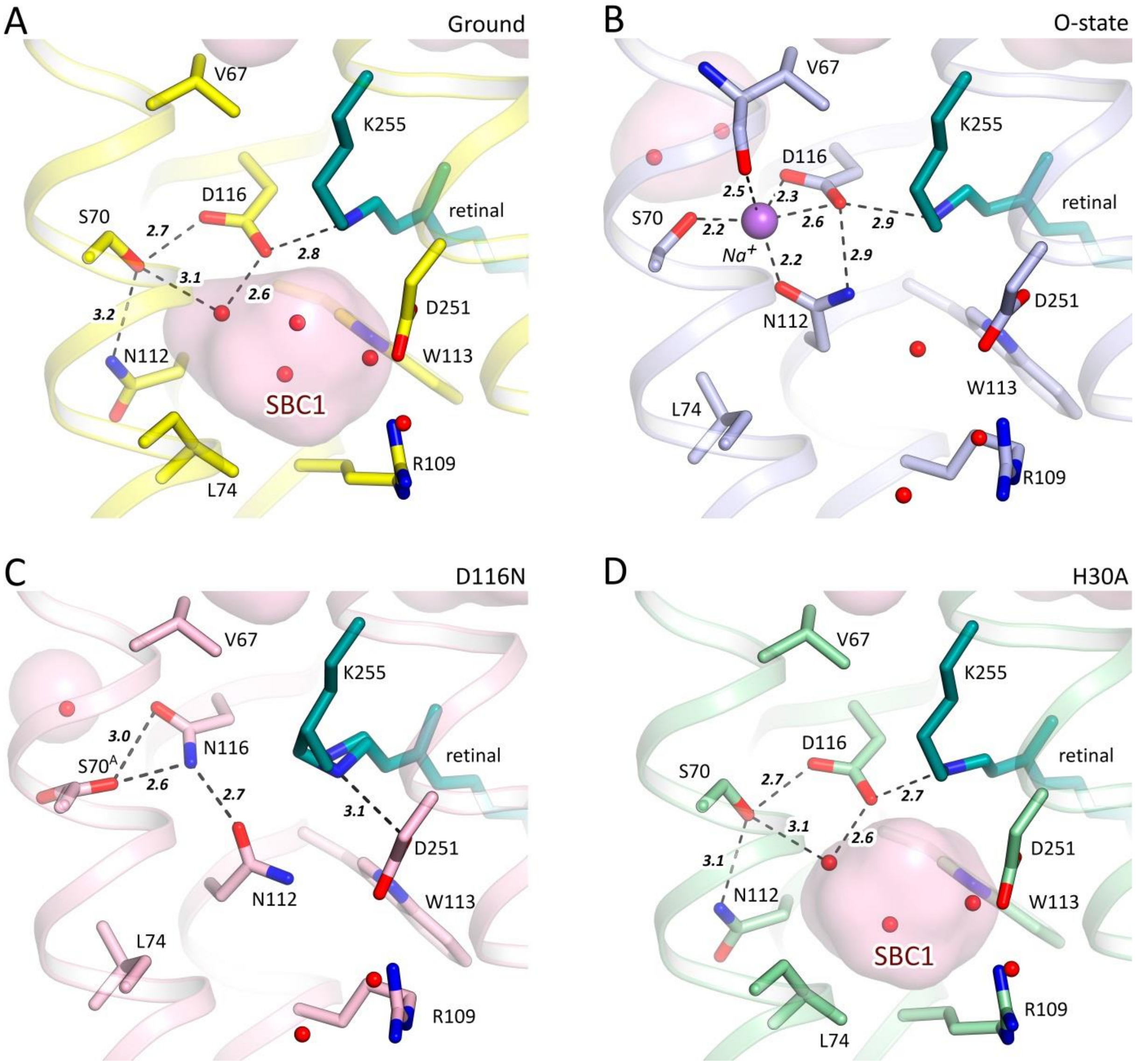

Supplementary Figure 13. RSB region and Na^+ binding site of KR2. **A.** The ground state of WT KR2 at pH 8.0 (PDB ID 6REW). **B.** The O-state of KR2 at pH 8.0 (present work). **C.** D116N mutant. **D.** H30A mutant. Cavities are calculated using HOLLOW[12] shown in pink and marked with red labels. Retinal cofactor is colored teal. Water molecules are shown with red spheres. Sodium ion is shown with a purple sphere. Hydrogen bonds involving S70, N112, D116, D251 and RSB are shown with black dashed lines. The lengths of the shown hydrogen bonds are shown with bold italic numbers and are given in Å. Helix A and SBC2 are hidden for clarity.

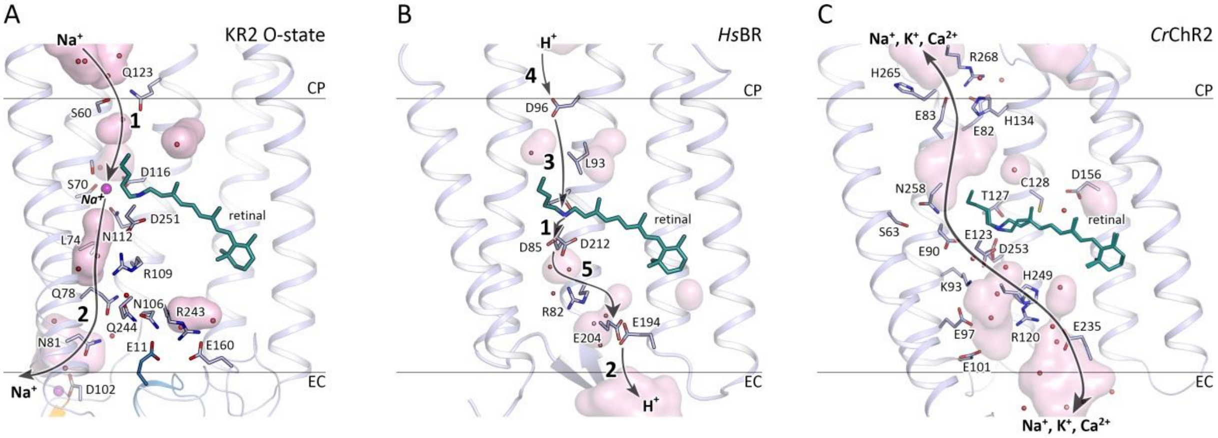

Supplementary Figure 14. Comparison of ion pathways in different classes of microbial rhodopsins. A. The O-state of the KR2 (present work). **B.** The ground state of the *Hs*BR[14] (PDB ID: 1C3W). **C.** Ground state of the *Cr*ChR2[15] (PDB ID: 6EID). Cavities are calculated using HOLLOW[12] and shown with pink surface. Gray lines indicate membrane hydrophobic/hydrophilic boundaries. Gray arrows indicate ion translocation pathways. In case of KR2 1 is for the Na+ uptake and 2 is for Na+ release. In case of *Hs*BR 1 is for proton translocation from the RSB to D85, 2 is for proton release from E194-E204 pair to extracellular space, 3 is for proton translocation from D96 to the RSB, 4 is for the D96 reprotonation from the cytoplasmic space and 5 is for the proton relocation from D85 to E194-E204 pair. In case of *Cr*ChR2 gray arrow indicate the ion pathway through the channel.

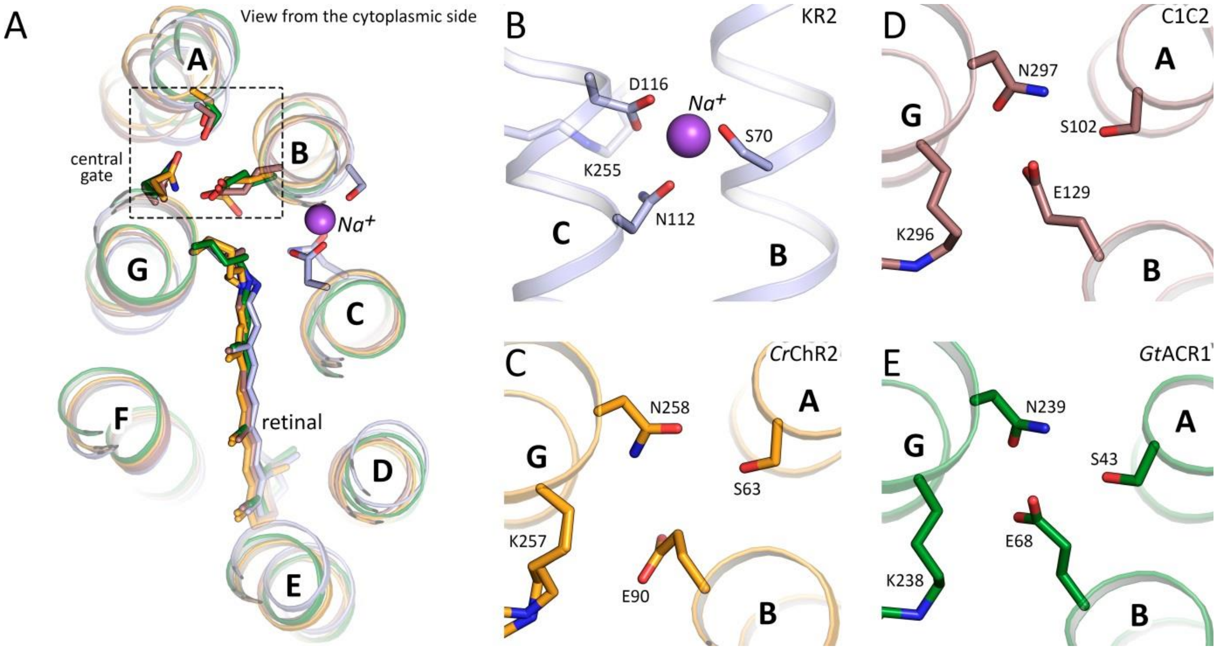

Supplementary Figure 15. Na^+ binding site of KR2 and central gates of channelrhodopsins. **A.** Structural alignment of KR2 (present work, blue), *Cr*ChR2 (PDB ID: 6EID, orange), C1C2 (PDB ID: 3UG9, brown) and *Gt*ACR1 (PDB ID: 6CSM, green). **B.** Transient sodium binding site in KR2. **C.** Central gate of *Cr*ChR2. **D.** Central gate of C1C2. **E.** Central gate of *Gt*ACR1.

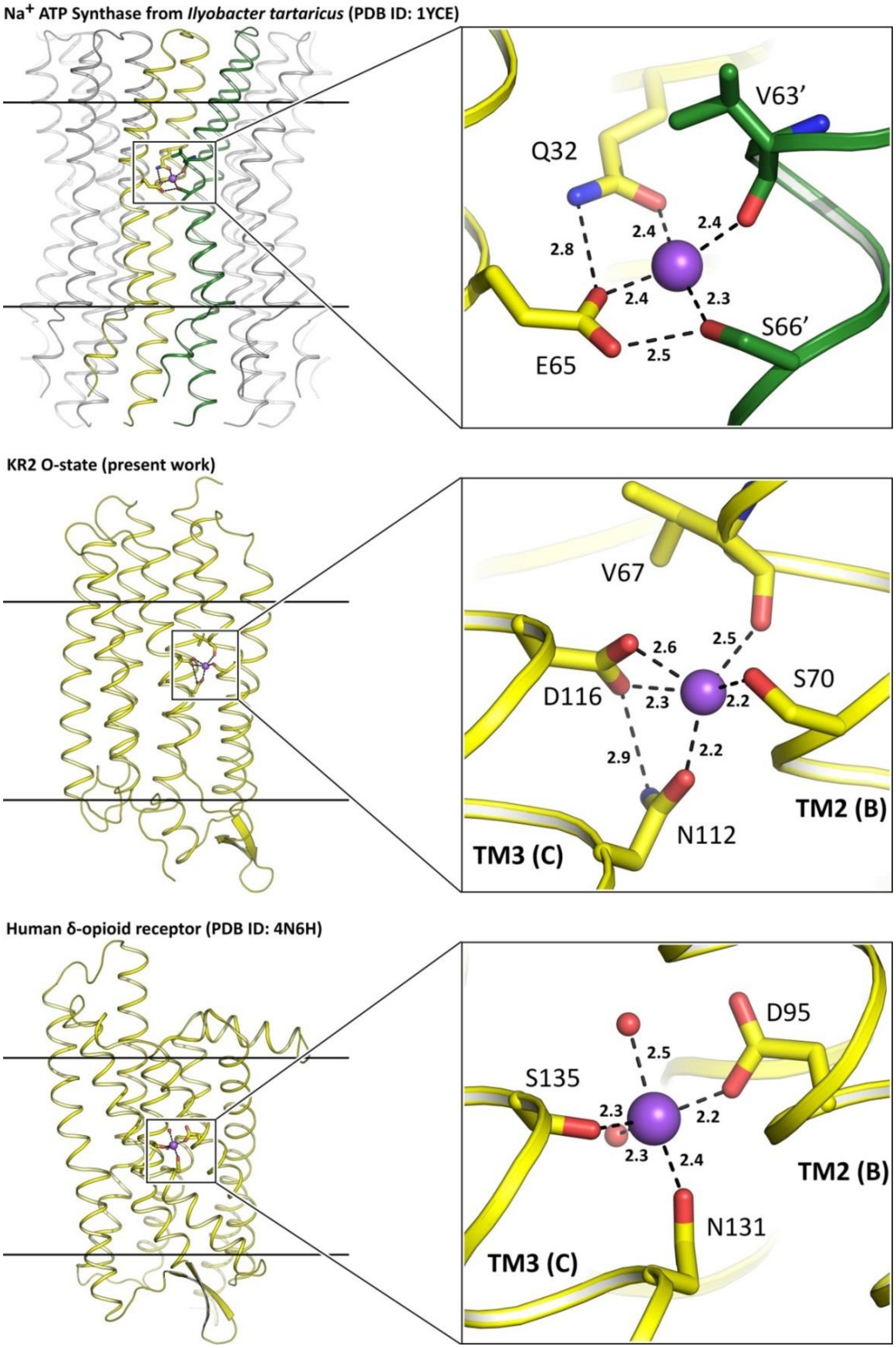

Supplementary Figure 16. Na^+ binding sites of different families of membrane proteins.

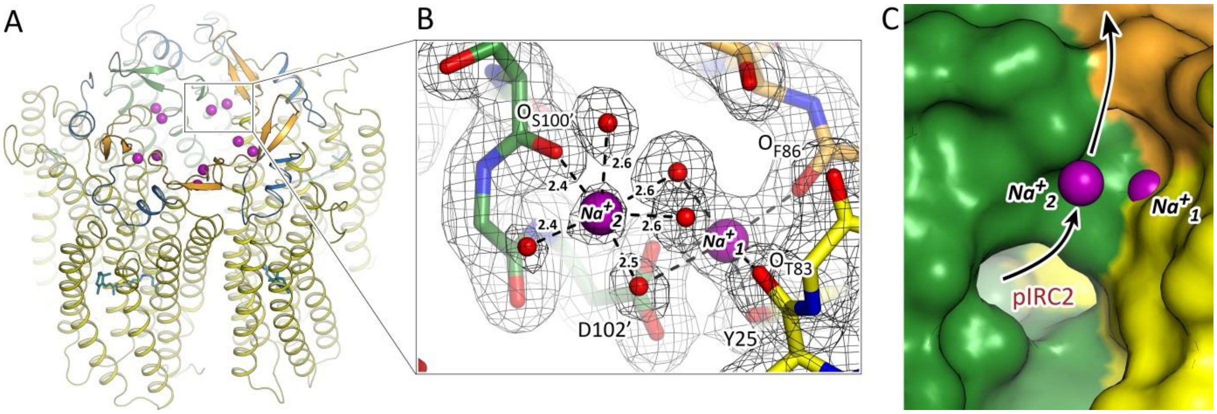

Supplementary Figure 17. Additional Na^+ identified at the KR2 interface in the ground state. A. Overall view of KR2 pentamer in the ground state from the extracellular side. Na^+ bound at the protein surface are shown with purple spheres. **B.** Zoomed-in view of the Na^+-binding sites. $2F_o$-F_c electron density maps around the Na^+ and interacting residues and water molecules are shown with black mesh and are contoured at the level of 1.2 σ. Distances between Na^+ and nearby oxygens are shown with black dashed lines. Distances are in Å. C. Surface of KR2 pentamer near Na^+. Putative ion-release cavity 2 (pIRC2) is labeled. Black arrows indicate putative relay pathway of the Na^+ release.

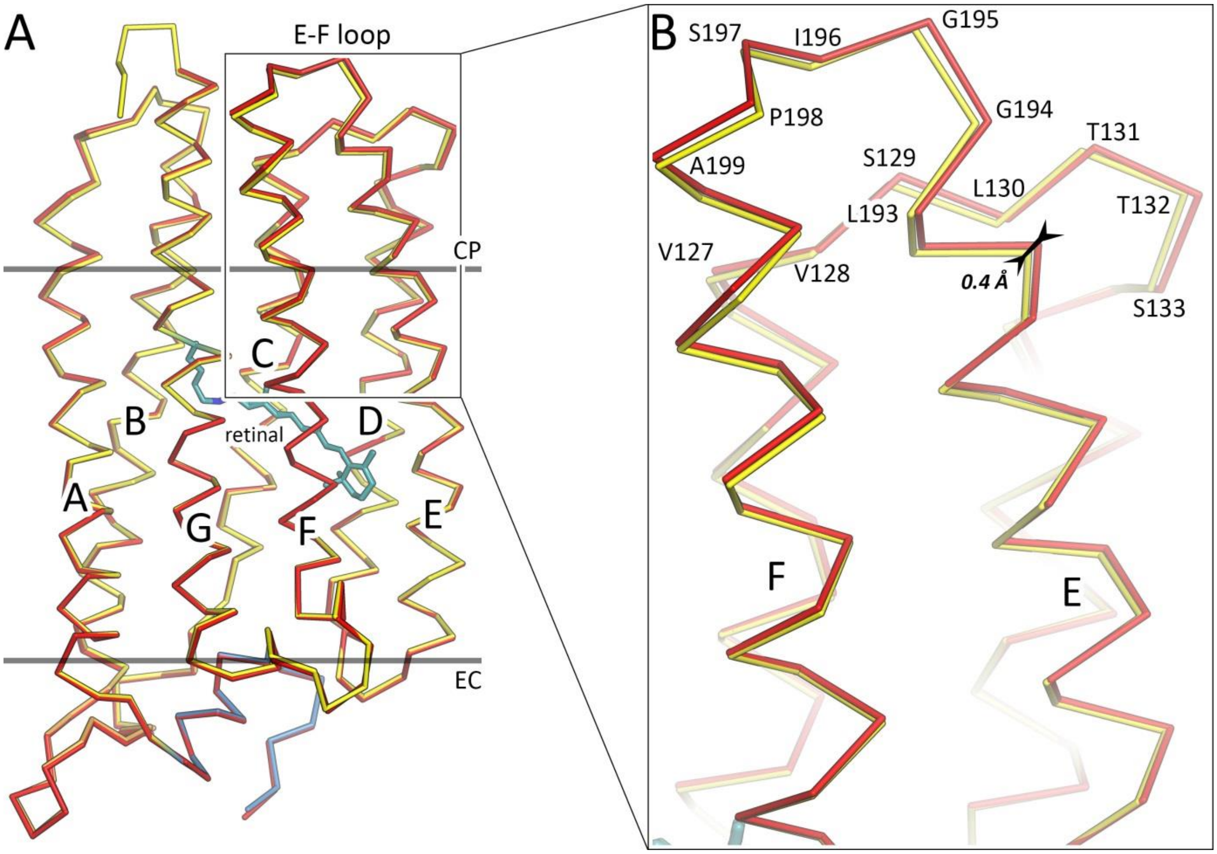

Supplementary Figure 18. Structural alignment of KR2 protomers at 100K and 293K. A. Overall alignment. N-terminal α-helix and N terminus are colored blue. BC loop, containing the β-sheet, is colored orange. Membrane core boundaries are shown with black lines. KR2 ground state model at 100 and 293K are colored yellow and red, respectively. Retinal cofactor is colored teal. **B.** Enlarged view of the most notable rearrangements in protomer backbone. Helices are indicated with bold capital letters. Residues comprising the E-F and C-D loops are labeled. Shift of the helix E is shown with the black arrow and the distance is indicated.

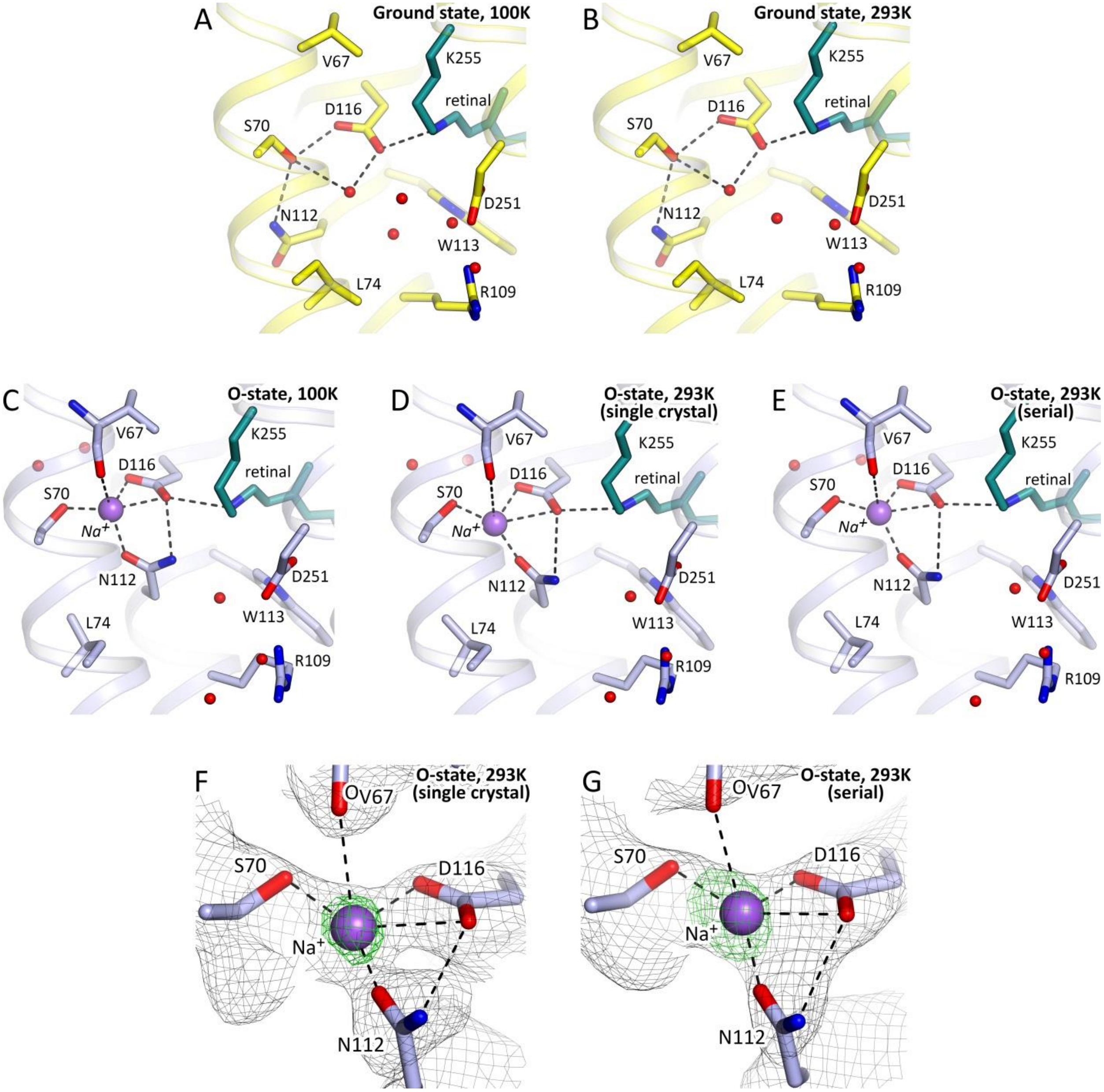

Supplementary Figure 19. RSB region of the ground and the O-states of KR2 at 100K and 293K. **A.** The ground state of KR2 at 100K (PDB ID: 6REW). **B.** The ground state of KR2 at 293K (present work). **C.** The O-state of KR2 at 100K (present work). **D.** The O-state of KR2 at 293K obtained using single-crystal crystallography (present work). **E.** The O-state of KR2 at 293K obtained using serial crystallography (present work). Retinal cofactor is colored teal. Water molecules are shown with red spheres. Na^+ is shown with a purple sphere. Hydrogen bonds are shown with black dashed lines. Helix A is hidden for clarity. **F, G.** Electron density maps of the Na^+ binding site in the O-state at 293K (black, $2F_o$-F_c at the level of 1.0σ; green, polder[11] difference maps omitting Na^+ are contoured at the level of 4.0σ and 3.0σ for the single-crystal (**F**) and serial crystallography (**G**), respectively).

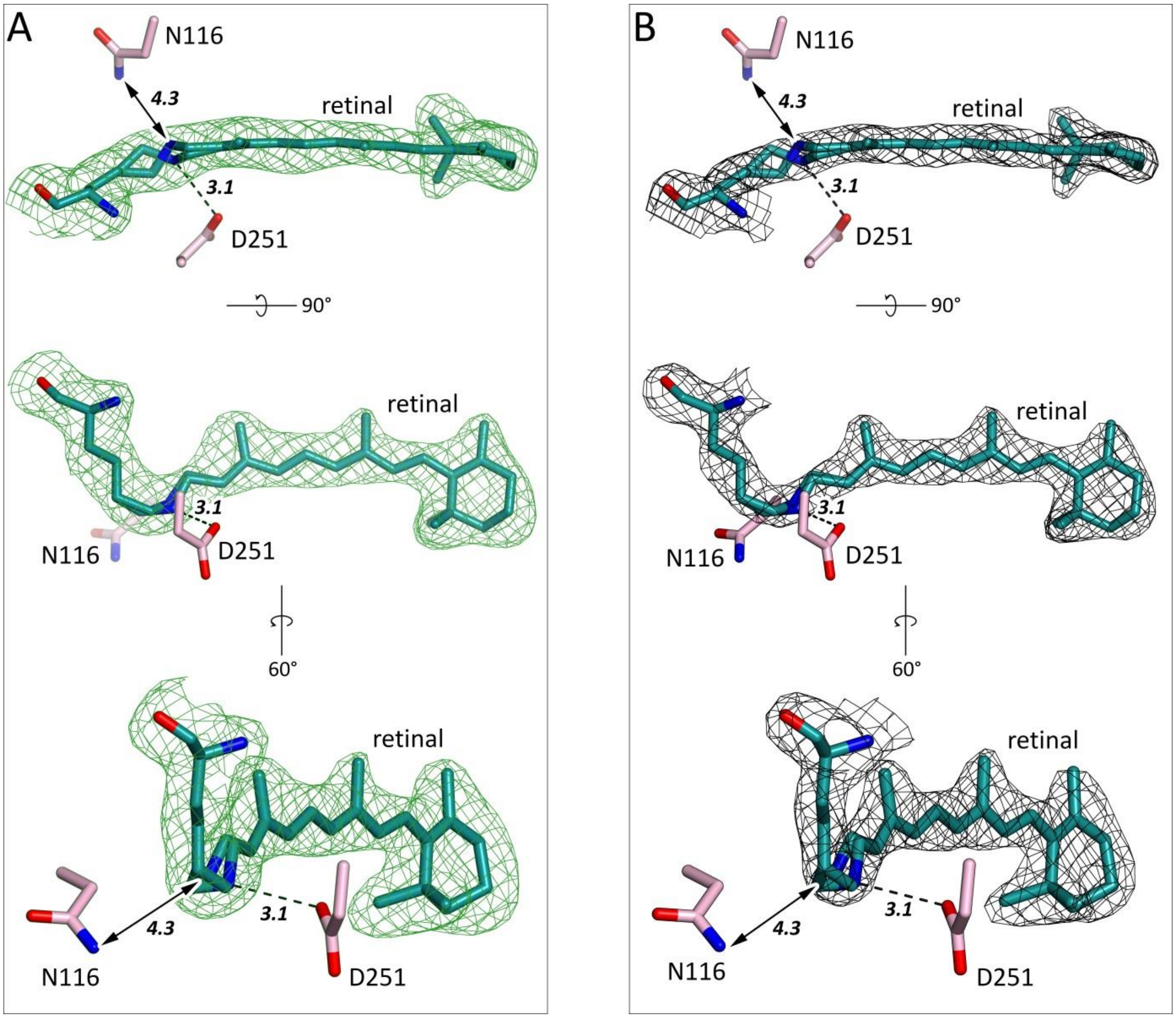

Supplementary Figure 20. Examples of electron densities of the KR2-D116N. A. Polder[11] maps for K255 and retinal cofactors of all five protomers of KR2-D116N structure. Maps are contoured at the level of 4.0 σ. **B.** $2F_o$-F_c electron density maps around K255 and retinal cofactor contoured at the level of 1.5 σ. Retinal and K255 are colored teal. Hydrogen bond between the RSB and D251 is shown with black dashed lines. The distance between the RSB and nearest atom of N116 is shown with black arrowed line. The lengths of the hydrogen bond and the distance are shown with bold italic numbers and are in Å.

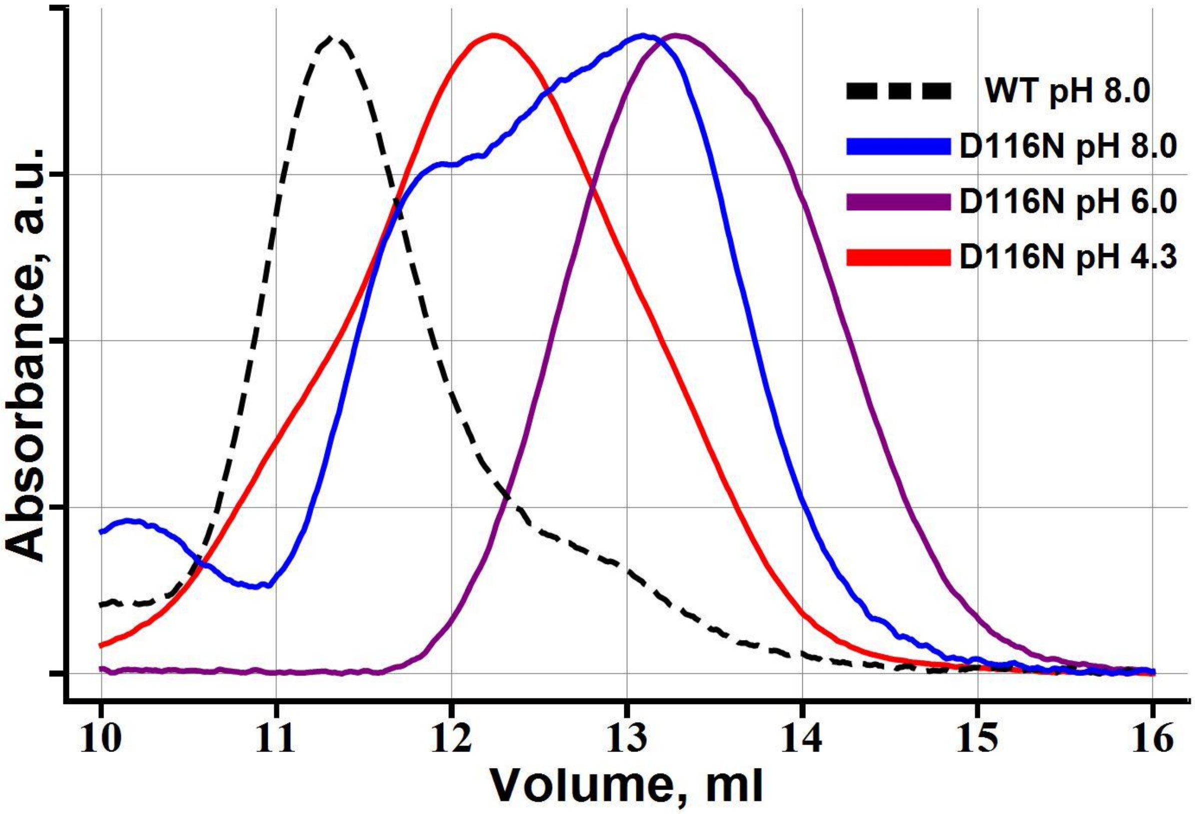

Supplementary Figure 21. Size exclusion chromatography profiles of D116N mutant of KR2. Protein with initial concentration of 70 mg/ml was dissolved in buffer solution containing 200 mM NaCl with 0.1% DDM to final concentration of 1 mg/ml and dialyzed against 100x volume of the buffer of needed pH with no less than 5 times substitution of the outer buffer solution during at least 72 hours.

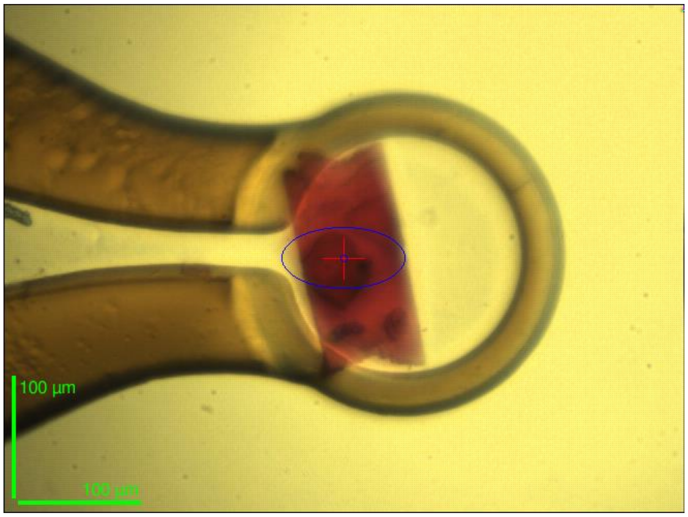

Supplementary Figure 22. Example of KR2 crystals used in present work. The mean size of the crystal is 200x100x30 μm^3. The same result of the O-state trapping procedure was reproduced with approximately 30 crystals of KR2, same as presented in the figure.

Supplementary Table 1. Data collection and refinement statistics.

	WT-100K Ground state	*WT-100K O-state*	*WT-293K Dark state*	*WT-293K Illuminated state (single crystal)*	*WT-293K Illuminated state (serial)*	*D116N-100K monomeric*	*D116N-100K pentameric*	*H30A-100K pentameric*
PDB ID	6YC3	6XYT	6YC2	6YC4	6YC0	6YBY	6YBZ	6YC1
pH	8.0	8.0	8.0	8.0	8.0	4.6	8.0	8.0
**Data collection**								
Space group	$C222_1$	$C222_1$	$C222_1$	$C222_1$	$C222_1$	I222	$C222_1$	$C222_1$
Number indexed/merged crystals	1/1	1/1	3/3	3/3	136656/38761	1/1	1/1	1/1
Cell dimensions								
a, b, c (Å)	131.87, 240.32, 135.51	131.16, 240.63, 135.04	135.15, 239.89, 138.35	134.92, 239.78, 138.35	135.21, 240.34, 138.37	40.89, 83.60, 233.83	131.34, 240.48, 135.41	131.09, 239.73, 135.13
α, β, γ (°)	90, 90, 90	90, 90, 90	90, 90, 90	90, 90, 90	90, 90, 90	90, 90, 90	90, 90, 90	90, 90, 90
Resolution (Å)	48.16-2.00 (2.03-2.00)	48.06-2.10 (2.14-2.10)	48.84-2.50 (2.55-2.50)	48.81-2.59 (2.65-2.59)	69.19-2.50 (2.54-2.50)	40.81-1.80 (1.84-1.80)	48.13-2.35 (2.39-2.35)	48.01-2.20 (2.24-2.20)
R_{merge} or R_{split} (%)	7.6 (174.9)	7.8 (276.0)	21.9 (236.5)	23.7 (213.1)	15.2 (150.4)	4.6 (100.0)	11.0 (192.5)	12.2 (209.9)
$I/\sigma I$	13.2 (1.2)	20.1 (1.1)	10.1 (1.2)	6.7 (1.1)	11.3 (0.7)	16.2 (1.7)	11.3 (1.0)	11.5 (0.9)
$CC_{1/2}$ (%)	99.9 (74.3)	99.8 (56.0)	99.8 (81.7)	99.4 (50.7)	98.8 (48.9)	99.9 (90.5)	99.9 (78.7)	99.9 (48.9)
Completeness (%)	99.9 (100.0)	99.8 (99.9)	100.0 (100.0)	99.6 (94.8)	100.0 (100.0)	99.8 (99.7)	99.9 (99.9)	98.9 (99.2)
Unique reflections	144,508 (7135)	123,865 (6133)	78,134 (4418)	69,594 (4259)	77,967 (7691)	37,771 (2191)	89,161 (4530)	106,450 (5261)
**Refinement**								
Resolution (Å)	50-2.0	50-2.1	50-2.5	50-2.6	50-2.7	20-1.8	50-2.35	50-2.20
No. reflections	135,234	101,547	74,363	59,103	58,999	35,822	74,883	91,343
R_{work}/R_{free} (%)	17.7/20.3	17.6/20.0	17.4/19.8	19.9/21.8	19.1/22.0	20.2/22.0	19.9/22.3	18.2/20.5
R.m.s deviations								
Protein bond lengths (Å)	0.0027	0.0027	0.0023	0.0012	0.0011	0.0054	0.0096	0.0019
Protein bond angles (°)	1.0671	1.0479	1.1192	1.0765	1.0751	0.7962	1.0876	1.0502
