# Supplementary material for: Molecular mechanism of light-driven sodium pumping
Source: Nat Commun. 2020 May 1;11:2137. doi: 10.1038/s41467-020-16032-y (PMC7195465; doi:10.1038/s41467-020-16032-y)
Supplement: Supplementary file 3 — Description of Additional Supplementary Information [file 41467_2020_16032_MOESM3_ESM.pdf]

Description of Additional Supplementary Files

File Name: Supplementary Movie 1
Description: Exemplary trajectory of Na+ release obtained using molecular dynamics.
